# Real-World Data on Halting Radiographic Progression with Antifibrotics in Connective Tissue Disease-Associated Interstitial Lung Disease: A Two-Center Study from Hungary

**DOI:** 10.3390/jcm15093539

**Published:** 2026-05-06

**Authors:** Zsuzsanna Gyetkó, Edit Végh, Dóra Nemes-Tömöri, Lilla Andó, Edit B. Nagy, Judit Maráz, Angéla Mikáczó, Anna Sárközi, Ildikó Horváth, István Takács, László Tóth, Gabriella Szűcs, Zoltán Szekanecz, Bernadett Bói, Gyöngyike Majai, Szilvia Szamosi

**Affiliations:** 1 Department of Rheumatology and Immunology, Faculty of Medicine, University of Debrecen, 4032 Debrecen, Hungary; gyetko.zsuzsanna@med.unideb.hu (Z.G.); vegh.edit@med.unideb.hu (E.V.); szucs.gabriella@med.unideb.hu (G.S.); szekanecz.zoltan@med.unideb.hu (Z.S.); 2 Division of Clinical Immunology, Institute of Internal Medicine, Faculty of Medicine, University of Debrecen, 4032 Debrecen, Hungary; nemes.tomori.dora@med.unideb.hu (D.N.-T.); mgyongyi@med.unideb.hu (G.M.); 3 Faculty of Medicine, University of Debrecen, 4032 Debrecen, Hungary; andolillabrigitta@mailbox.unideb.hu; 4 Department of Radiology, Faculty of Medicine, University of Debrecen, 4032 Debrecen, Hungary; nagy.edit@med.unideb.hu (E.B.N.); maraz.judit@med.unideb.hu (J.M.); 5 Department of Pulmonology, Faculty of Medicine, University of Debrecen, 4032 Debrecen, Hungary; mikaczo.angela@med.unideb.hu (A.M.); sarkozi.anna@med.unideb.hu (A.S.); horvath.ildiko@med.unideb.hu (I.H.); 6 Department of Surgery, University of Debrecen, 4032 Debrecen, Hungary; takacs.istvan@med.unideb.hu; 7 Department of Pathology, University of Debrecen, 4032 Debrecen, Hungary; tothlasz@med.unideb.hu; 8 Department of Public Health and Epidemiology, Faculty of Medicine, University of Debrecen, 4028 Debrecen, Hungary; boi.bernadett@med.unideb.hu

**Keywords:** realworld, CTDILD, nintedanib, fibrosis, radiography, progression, lungfunction

## Abstract

**Background/Objectives**: Connective tissue disease-associated interstitial lung disease (CTD-ILD) is linked to substantial morbidity and mortality. While nintedanib (NTB) slows lung function decline in progressive pulmonary fibrosis (PPF), real-world data—particularly regarding radiographic outcomes—remain limited. We aimed to evaluate the real-world effectiveness and tolerability of antifibrotic therapy—predominantly NTB—on radiographic and functional outcomes in a Hungarian CTD-ILD cohort. **Methods**: We conducted a retrospective observational cohort study including 72 patients with progressive CTD-ILD who initiated antifibrotic therapy at two Hungarian tertiary centers between January 2021 and June 2025. The primary endpoint was the proportion of patients without significant radiographic progression at 6–12 months, based on blinded assessment of paired high-resolution computed tomography (HRCT) scans by two thoracic radiologists. Secondary endpoints included changes in forced vital capacity (FVC) and diffusing capacity for carbon monoxide (DLCO) at 6 and 12 months, safety and tolerability, and correlations between lung function and disease-related factors. **Results**: The cohort comprised systemic sclerosis–ILD (*n* = 25), rheumatoid arthritis–ILD (*n* = 23), and other CTD-ILD (*n* = 24). Radiographic stability was observed in 65.8–78.9% of patients, with improvement most commonly seen in ground-glass opacities, while traction bronchiectasis remained largely unchanged. Radiographic disease extent showed the strongest inverse correlation with baseline FVC and DLCO (*p* < 0.05). Significant improvements in FVC and DLCO were observed at 6 and 12 months (*p* < 0.001). Antifibrotic therapy was well tolerated, including in combination with immunosuppressive treatment. **Conclusions**: These real-world data support the effectiveness and safety of NTB in PPF–CTD-ILD and highlight radiologic disease burden as a key determinant of functional impairment.

## 1. Introduction

Patients with CTD-ILD experience a substantial disease burden, including markedly reduced quality of life, high levels of disability, and considerable mortality [1]. Pulmonary involvement may develop across a spectrum of CTDs, including SSc, RA, idiopathic inflammatory myopathies (IIM), Sjögren’s disease (SjD), mixed connective tissue disease (MCTD), systemic lupus erythematosus (SLE), anti-neutrophil cytoplasmic antibody (ANCA)-associated vasculitis (AAV), and undifferentiated connective tissue disease (UCTD) [2]. While CTD is typically diagnosed before ILD onset, a significant proportion of patients present with ILD as the initial manifestation [3].

Although pathophysiologic and clinical features share similarities, the prevalence, risk factors, characteristic chest CT patterns, and rate of disease progression vary significantly by disease subtype. A recent meta-analysis has shown the highest ILD prevalence rates in MCTD (mean: 52%; range: 39–72%), SSc (mean: 47%; range: 47–50%), and IIM (mean 41%; 33–50%), which stands in contrast to the significantly lower rates observed in SjD (mean: 17%; range: 12–21%), RA (mean: 11%; range: 7–15%), and SLE (mean: 6%; range: 3–10%) [4].

Mortality risk is particularly high in individuals who develop the PPF phenotype, which is an indicator of a poor prognosis [5]. Several criteria are used to define CTD-ILD progression, the most frequent of which was adopted from the INBUILD trial. These progression criteria are based on a combination of lung function decline, worsening of respiratory symptoms, and/or radiographic progression of lung fibrosis on HRCT [6]. The prevalence of PPF in patients with CTD-ILD has been reportedly 23–38% [7], with the highest progression rates observed in RA-ILD (40%) and SSc-ILD (32%), compared to SjD-ILD (24%) and IIM-ILD (16%) [8]. Standardized recommendations, such as the 2025 expert consensus from the European Respiratory Society (ERS) and the European Alliance of Associations for Rheumatology (EULAR) [9], provide a critical framework for the multidisciplinary management of these patients, where the rheumatologist acts as the conductor, coordinating care with pulmonologists and radiologists. These recommendations outline tools for defining disease progression and rely on structured progression criteria similar to those used in INBUILD [6]. While the primary endpoint of these trials was the annual rate of decline in FVC, radiographic assessment by HRCT is a critical component of ILD diagnosis and monitoring. Halting the progression of fibrosis on HRCT is a key therapeutic goal [10]. Real-world evidence is essential to validate trial results in more heterogeneous, unselected populations and to assess outcomes like radiographic stability in routine clinical practice [11]. Data on the use of NTB for CTD-ILD in Central and Eastern European populations are scarce. This study aims to describe the real-world experience of two Hungarian tertiary care centers, with a specific focus on drug efficacy in halting radiographic progression in patients with CTD-ILD.

## 2. Materials and Methods

### 2.1. Study Design

A retrospective, observational cohort study was conducted using data from consecutive patients with CTD-ILD who initiated antifibrotic therapy (nintedanib or pirfenidone) at two tertiary care centers in Hungary (Department of Rheumatology and Immunology, University of Debrecen, and Division of Clinical Immunology, Department of Internal Medicine, University of Debrecen) between January 2021 and June 2025.

### 2.2. Patient Characteristics and Inclusion Criteria

The diagnosis of CTD was established by a rheumatologist according to internationally accepted classification criteria (1980 LeRoy criteria for UCTD [12]; 2013 American College of Rheumatology (ACR)/EULAR criteria for SSc [13]; 2010 ACR/EULAR criteria for RA [14]; 2017 EULAR/ACR criteria for IIM [15]; 1987 Alarcón-Segovia criteria for MCTD [16]; 2016 ACR/EULAR criteria for primary Sjögren’s syndrome [17]; 2022 ACR/EULAR criteria for AAV [18]; 2015 ERS/American Thoracic Society (ATS) research criteria for interstitial pneumonia with autoimmune features (IPAF) [19]). The diagnosis and subtype of CTD-ILD were confirmed by multidisciplinary discussion (MDD) involving a pulmonologist, rheumatologist/immunologist, radiologist, pathologist, and thoracic surgeon, based on clinical, serological, and HRCT findings. The indication for NTB was the presence of PPF, defined according to the 2022 ATS/ERS/Japanese Respiratory Society (JRS)/Latin American Thoracic Association (ALAT) international guidelines [20]. In line with current clinical practice, NTB was considered the preferred antifibrotic agent and was initiated in the absence of contraindications [10]. Pirfenidone (PFD) was reserved for the very small proportion of patients in whom NTB was contraindicated, and its use in these cases was off-label [21]. NTB was prescribed according to the manufacturer’s prescribing information and national reimbursement guidelines. Absolute contraindications for NTB initiation included: (1) moderate-to-severe hepatic impairment (Child-Pugh B or C); (2) pregnancy or breastfeeding; (3) known hypersensitivity to nintedanib, peanut, soya, or any excipient of the drug; and (4) a history of severe gastrointestinal intolerance to prior antifibrotic therapy (either NTB or pirfenidone) that led to permanent treatment discontinuation. Relative contraindications, assessed on a case-by-case basis, were recent major surgery (within 4 weeks), active bleeding diathesis, or concurrent use of strong CYP3A4 inducers (e.g., rifampin, carbamazepine). Patients were required to have met at least two of the following three criteria of disease progression within the 12 months prior to treatment initiation: (1) physiologic progression, defined as an absolute decline in FVC% predicted of ≥5% and/or an absolute decline in DLCO % predicted of ≥10%; (2) radiologic progression on HRCT, as confirmed by an expert thoracic radiologist, evidenced by an increased extent of fibrosis (reticulation, honeycombing), new or worsening traction bronchiectasis, or increased lobar volume loss; and (3) worsening respiratory symptoms. Additionally, in accordance with the Hungarian national reimbursement algorithm, eligibility for NTB /PFD required a radiographically confirmed fibrotic lung involvement of ≥20% on HRCT, a criterion that was met by all included patients. Inclusion criteria were: (1) age ≥ 18 years; (2) a confirmed MDD diagnosis of CTD-ILD; (3) initiation of NTB or PFD therapy; and (4) availability of at least one follow-up clinical assessment (≥6–24 months) after treatment initiation. Patients were excluded if clinical data were insufficient for analysis or if follow-up was less than 6 months.

### 2.3. Data Collection

Demographic, clinical, serological, and therapeutic data and pulmonary function tests (PFTs) were extracted from electronic medical records. Lung function tests were performed using whole-body plethysmography (PDT-111/pd, Piston Medical, Budapest, Hungary). Manufacturer-provided algorithms were used to record raw pulmonary function data and to calculate percentages of predicted values. Spirometry and diffusing capacity measurements were conducted in accordance with international standards. The collected variables included age, sex, smoking history, environmental and occupational exposures, and disease-related characteristics such as the specific CTD diagnosis, duration of CTD and ILD, and autoantibody profile. Disease activity was assessed using standardized indices: for RA, the disease activity score in 28 joints using C-reactive protein (DAS28-CRP); and for SSc, the modified Rodnan skin score (mRSS) was determined by a rheumatologist using the 17-site method (range 0–51). The following PFT parameters were collected at baseline (within 3 months prior to NTB/PFD initiation) and at follow-up intervals (6 and 12 months post-initiation): FVC, expressed in absolute liters (L) and as a percentage of predicted values (% pred), and DLCO, expressed as a percentage of predicted values (% pred). The availability of these measurements at each time point was documented. Radiographic assessment was based on baseline and follow-up HRCT scans (performed 6–12 months after treatment initiation), which were independently evaluated by two ILD-experienced radiologists who were blinded to clinical data but aware of scan chronology, using the General Electric HealthCare Advanced Workstation Server (version 3.2, extension 3.0). They assessed the following parameters: ILD pattern (i.e., usual interstitial pneumonia, non-specific interstitial pneumonia, organizing pneumonia, and fibrotic hypersensitivity pneumonitis), presence and extent of honeycombing, total lung volume (TLV), and overall ILD extent (% of lung involvement). Radiographic progression was defined as a clear increase in the extent of reticulation, honeycombing, or traction bronchiectasis [10,22]. Patients were subsequently classified into one of five categories based on the therapeutic response: (1) no change; (2) minimal improvement; (3) moderate/significant improvement; (4) minimal worsening; and (5) moderate/significant worsening. Information on antifibrotic therapy (NTB or PFD), treatment duration, dose adjustments, concomitant immunosuppressive treatment, and details of prior immunosuppressive therapies were also recorded. Safety and tolerability were evaluated by assessing the incidence of adverse events (AEs), reasons for dose reduction, and causes of permanent treatment discontinuation.

### 2.4. Outcome Measures

Primary endpoint: The primary endpoint was the proportion of patients without significant radiographic progression on HRCT after 6–12 months of antifibrotic therapy, as assessed by two independent thoracic radiologists.

Secondary endpoints: Changes in PFTs, specifically FVC % predicted and DLCO % predicted, from baseline to 6 and 12 months; safety and tolerability of antifibrotic therapy, including rates of dose reduction and permanent discontinuation; and correlation between baseline PFTs and disease-specific factors (e.g., ILD extent, disease duration).

### 2.5. Statistical Analysis

The analysis was performed using R (version 4.3.1, R Foundation for Statistical Computing, Vienna, Austria) in RStudio (version 2024.09.0, Posit Software, PBC, Boston, MA, USA) with the gtsummary [23] and ggplot2 [24] packages. Descriptive statistics were used to characterize the cohort. Categorical variables are presented as frequencies (*n*) and percentages (%), and comparisons were made using the Chi-square test or Fisher’s exact test, as appropriate. Continuous variables were tested for normality using the Shapiro–Wilk test. For normally distributed variables, data are presented as mean and standard deviation (SD), whereas for non-normally distributed variables, median and interquartile range (IQR) were calculated. For comparisons of independent samples, one-way ANOVA was applied for parametric variables and the Kruskal–Wallis H test for non-parametric variables. For paired or related samples, repeated-measures ANOVA (parametric) or the Friedman test (non-parametric) was used. Interrater reliability between the two radiologists was assessed separately for categorical and continuous variables using complete-case paired readings only. For categorical variables, Cohen’s kappa was calculated between the two raters. For continuous variables, a two-way absolute-agreement single-measure intraclass correlation coefficient (ICC) was calculated. Reliability was evaluated separately at each HRCT scan time point. Associations between pre-treatment pulmonary function parameters and disease-specific variables were assessed using Spearman’s rank correlation coefficient (rs) for two continuous variables, and point-biserial correlation (rpb) for relationships between a continuous and a dichotomous variable. All statistical tests were two-sided, and a *p*-value < 0.05 was considered statistically significant.

## 3. Results

### 3.1. Patient Characteristics

Our study cohort comprised 72 patients with a confirmed diagnosis of CTD-ILD (Table 1). The population was categorized into three principal groups for comparative analysis: SSc-ILD (*n* = 25, 34.7%), RA-ILD (*n* = 23, 31.9%), and a composite group of other CTD-ILDs (*n* = 24, 33.3%). The latter included patients with IPAF (*n* = 6, 8.3%), IIM (*n* = 5, 6.9%), overlap syndromes (*n* = 5, 6.9%), AAV (*n* = 3, 4.2%), SjD (*n* = 3, 4.2%), and MCTD (*n* = 2, 2.7%), as detailed in Supplementary Material, Table S1.

Baseline characteristics of the entire cohort and these three groups are detailed in Table 1.

The groups were well-matched for age and sex distribution. The median age for this cohort was 64.5 years (IQR 57.0–68.0), with females comprising 70.8% of patients. However, smoking history emerged as a significant differentiating factor (*p* < 0.001). Patients with RA-ILD had a substantially higher prevalence of current (30.4%) and past (43.5%) smoking, whereas the SSc-ILD and other CTD-ILD groups were predominantly never-smokers (72.0% and 70.8%, respectively). Significant differences were observed in the chronology of CTD and ILD diagnoses. The duration of the underlying CTD was longest in the SSc-ILD group (median 13.0 years), which was significantly longer than in other CTD-ILD groups (median 5.5 years, *p* = 0.044). Furthermore, the duration of known ILD—calculated from the date of ILD diagnosis to the initiation of antifibrotic therapy—also varied significantly across the groups (*p* = 0.019). Patients with SSc-ILD had considerably longer documented ILD history (median 6.0 years) prior to antifibrotic initiation compared to those with RA-ILD (median 1.0 years). Autoantibody profiles reflected the distinct serological signatures of each CTD (Table 1). As expected, RF and anti-CCP antibodies were highly prevalent in the RA-ILD group (78.2% and 69.5%, respectively), while anti-Scl70 (anti-topoisomerase I) was detected almost exclusively in SSc-ILD (60.0%). Anti-Ro52 (tripartite motif-containing protein 21) antibodies were most frequent in the other CTD-ILD group (37.5%), though this difference did not reach statistical significance. Anti-Jo1 (anti-histidyl-tRNA synthetase) positivity was confined to the other CTD-ILD group (16.7%, *p* = 0.005). Regarding disease activity indices, among RA-ILD patients the median DAS28-CRP was 3.2 (IQR 2.4–4.9), indicating low-to-moderate disease activity at baseline. In SSc-ILD patients, the median mRSS was 8.0 (IQR 4.0–12.0), reflecting mild-to-moderate skin involvement. Distinct patterns of immunosuppressive therapy were evident, reflecting the differing treatment paradigms for the underlying connective tissue diseases (Table 1). Methotrexate (MTX) and rituximab (RTX) were the dominant agents in RA-ILD (65.2% and 39.1%, respectively), whereas mycophenolate mofetil (MMF) and cyclophosphamide (CYC) were the cornerstones of therapy for SSc-ILD (48.0% and 52.0%) and the other CTD-ILD group (37.5% and 58.3%). All between-group differences were statistically significant (*p* < 0.05). In contrast to the predominantly sequential monotherapy observed in prior treatment histories, a shift toward concomitant combination regimens was noted following the initiation of NTB. Specifically, 14.3% (5/35) of all patients receiving active concomitant therapy were treated with a combination of a biologic agent (RTX or tocilizumab (TCZ)) and MMF. Combination regimens were most common in the other CTD-ILD subgroup, where they comprised 33.3% (5/15) of all active treatments. Across all groups, a similar proportion of patients (37.5–44.0%) were not receiving any immunosuppressive therapy at the time of evaluation (*p* = 0.890).

### 3.2. Changes in Pulmonary Function Following Antifibrotic Therapy

**Figure 1:**
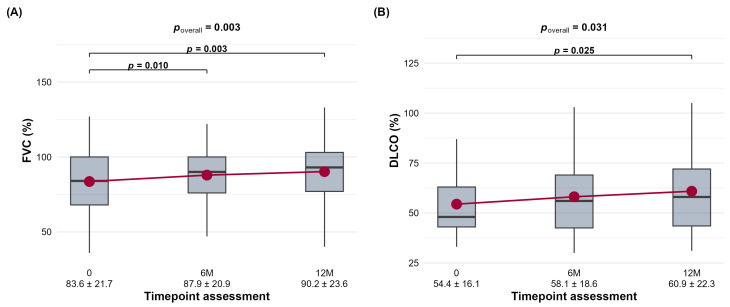
The distribution of forced vital capacity (FVC,%) (**A**) and diffusing capacity for carbon monoxide (DLCO,%) (**B**) in the overall CTD-ILD cohort at baseline (0), 6 months (6M), and 12 months (12M) is shown.

For the entire CTD-ILD cohort, a significant improvement in FVC was observed at both the 6-month (6M) (*p* < 0.001) and 12-month (12M) (*p* < 0.001) time points compared to their respective reference values (Table 2) (Figure 1A). Similarly, DLCO showed significant improvement at 6M (*p* < 0.001) and 12M (*p* < 0.001) (Figure 1B).

**Figure 2:**
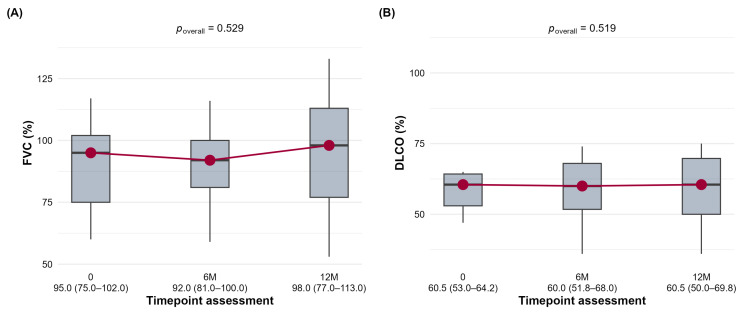
The distribution of forced vital capacity (FVC,%) (**A**) and diffusing capacity for carbon monoxide (DLCO, %) (**B**) in the SSc-ILD cohort at baseline (0), 6 months (6M), and 12 months (12M) is shown.

**Figure 3:**
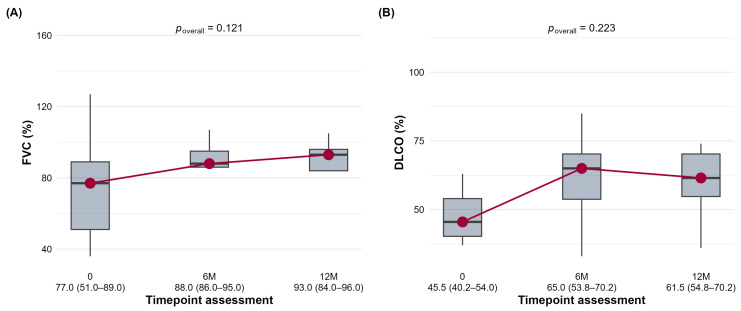
The distribution of forced vital capacity (FVC,%) (**A**) and diffusing capacity for carbon monoxide (DLCO,%) (**B**) in the RA-ILD cohort at baseline (0), 6 months (6M), and 12 months (12M) is shown.

**Figure 4:**
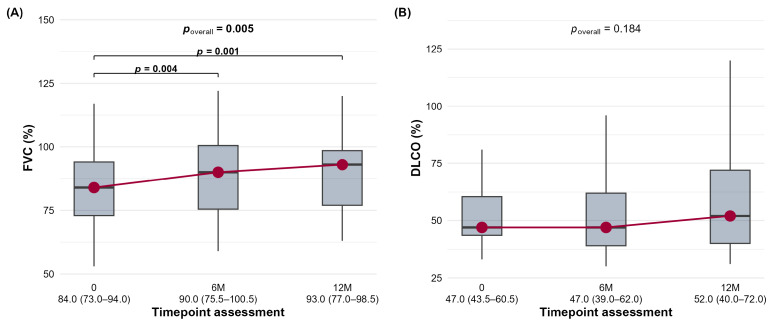
The distribution of forced vital capacity (FVC,%) (**A**) and diffusing capacity for carbon monoxide (DLCO,%) (**B**) in the other CTD-ILD cohort at baseline (0), 6 months (6M), and 12 months (12M) is shown.

When assessing the magnitude of change, the majority of patients across all groups remained stable (FVC: 58.9% at 6M, 64.7% at 12M; DLCO: 61.8% at 6M, 81.3% at 12M). However, a substantial proportion of patients experienced clinically significant improvement (≥5% FVC increase or ≥10% DLCO increase), particularly at the 6-month mark (FVC: 33.9%; DLCO: 27.3%). Analysis by disease subgroup revealed distinct patterns, further illustrated by the paired data in Figure 2, Figure 3 and Figure 4. However, in the SSc-ILD group, the mean FVC and DLCO values appeared stable, a significant within-group improvement in FVC was noted at 6 months (*p* < 0.001) (Figure 2A), and a significant change in DLCO (Figure 2B) was seen at both time points (*p* = 0.002 and *p* = 0.044). The distribution of FVC categories shifted favorably, with the proportion of patients with an FVC > 80% increasing from 59.1% at baseline to 76.5% at 6 months. The overwhelming majority of patients (82.4% at 6M) were categorized as stable.

The RA-ILD group demonstrated the most pronounced numerical improvements. A significant increase in FVC was seen at 6 months (*p* = 0.047) (Figure 3A), and a highly significant improvement in DLCO (Figure 3B) was observed at both 6 and 12 months (*p* = 0.015 and *p* = 0.002). Notably, more than half (52.9%) of RA-ILD patients with available data exhibited a >5% improvement in FVC at 6 months, representing the highest rate of improvement observed across all cohorts.

In the other CTD groups, a significant within-group improvement in FVC was sustained from baseline to 6 months (*p* = 0.062, a strong trend) and from 6 to 12 months (*p* = 0.022) (Figure 4A). DLCO showed a significant improvement at 12 months (*p* < 0.001) (Figure 4B). In addition, this group had a notable proportion of patients exhibiting favorable changes (FVC: 31.8% at 6M; DLCO: 26.1% at 6M).

### 3.3. Radiographic Patterns and Response to Therapy

**Figure 5:**
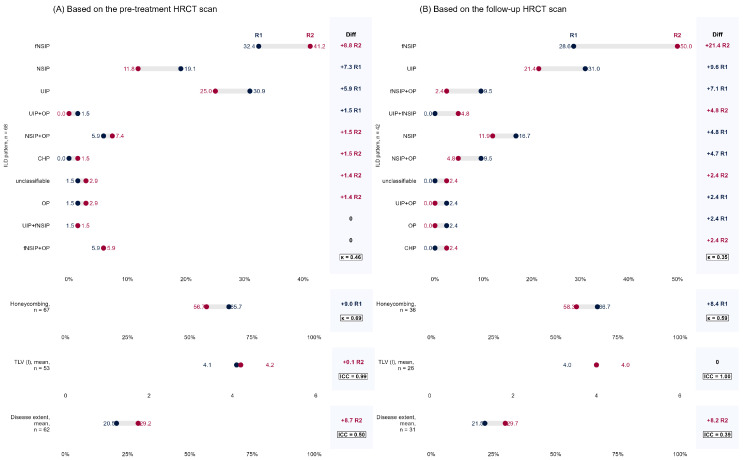
The distributions of high-resolution computed tomography (HRCT) patterns and radiological characteristics in the CTD-ILD cohort at pre-treatment (**A**) and follow-up (**B**) assessments are shown, including pattern classification, honeycombing, total lung volume (TLV), and disease extent. Abbreviations: HRCT—high-resolution computed tomography; ILD—interstitial lung disease; NSIP—non-specific interstitial pneumonia; fNSIP—fibrotic non-specific interstitial pneumonia; UIP—usual interstitial pneumonia; OP—organizing pneumonia; CHP—chronic hypersensitivity pneumonitis; TLV—total lung volume; R1—radiologist 1; and R2—radiologist 2.

Baseline HRCT scans, independently evaluated by two radiologists (R1 and R2), demonstrated the expected spectrum of ILD patterns characteristic of CTD-ILD (Figure 5). As illustrated in Figure 5A, the most common findings included ground-glass opacities (GGO), reticulation, and traction bronchiectasis. The distribution of patterns like honeycombing (HC) and the visual assessment of TLV and disease extent showed a high degree of qualitative concordance between the two radiologists, with minor discrepancies noted in the “Diff.” (Difference) plots. The assessment of radiographic response following treatment is summarized in Table 3. The analysis revealed a generally stable or improved disease course in most patients.

When evaluating the overall ILD, the most frequent outcome was “no change,” reported in 34.2% (R1) to 47.4% (R2) of patients (Figure 5B). A combined category of “minimal” to “moderate/marked improvement” was observed in 36.8% (R1) and 31.6% (R2) of patients. The rate of radiographic progression (minimal or moderate/marked worsening) was reported in 28.9% (R1) and 21.1% (R2) of cases. More detailed analysis showed that GGO exhibited the greatest improvement, with 34.5% (R1: 13.8% minimal + 20.7% marked) to 37.9% (R2: 20.7% minimal + 17.2% marked) of patients demonstrating minimal to marked radiographic improvement. Traction bronchiectasis was largely stable, with no change reported in 67.6% (R1) to 75.7% (R2) of patients. Worsening was infrequently observed. A consistent trend was observed across all assessments, with R2 tending to report a higher frequency of “no change,” while R1 more frequently identified minor changes, both improvement and worsening. For instance, in the overall therapeutic response, R2 classified 13.2% more patients as having “no change” compared to R1 (Figure 5B). Interrater agreement for treatment response varied by CT parameter. For GGO, κ = 0.32 (fair, *n* = 29); bronchiectasis, κ = 0.11 (slight, *n* = 37); and overall CT response, κ = 0.36 (fair, *n* = 38). ILD pattern agreement: pre-treatment κ = 0.46 (moderate, *n* = 68), and follow-up κ = 0.35 (fair, *n* = 42). Honeycombing: pre-treatment κ = 0.69 (substantial, *n* = 67), and follow-up κ = 0.59 (moderate, *n* = 36). TLV showed excellent agreement (pre-treatment ICC = 0.99, *n* = 53; follow-up ICC = 1.00, *n* = 26). ILD extent agreement: pre-treatment ICC = 0.50 (moderate, *n* = 62), and follow-up ICC = 0.39 (weak, *n* = 31).

**Figure 6:**
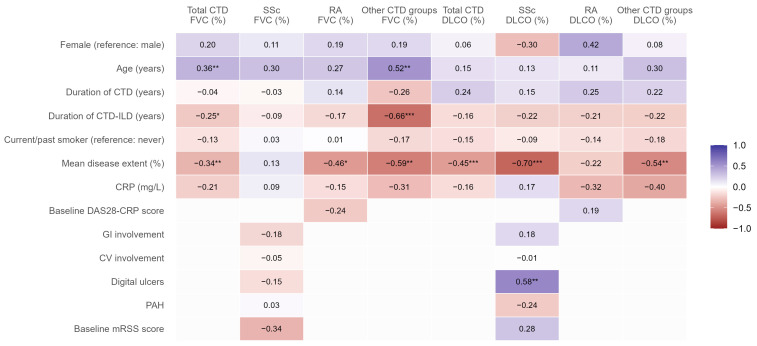
Correlation between clinical characteristics and lung function parameters in CTD-ILD patients. Heat map illustrating the correlation coefficients between demographic and clinical variables and lung function parameters, including forced vital capacity (FVC, %) and diffusing capacity for carbon monoxide (DLCO, %), in the overall CTD-ILD cohort as well as in systemic sclerosis (SSc), rheumatoid arthritis (RA), and other CTD subgroups. Positive correlations are shown in blue and negative correlations in red, with color intensity proportional to the strength of the association. Statistically significant correlations are indicated by asterisks (* *p* < 0.05; ** *p* < 0.01; *** *p* < 0.001). Abbreviations: CTD—connective tissue disease; CTD-ILD—connective tissue disease-associated interstitial lung disease; DAS28-CRP—28 joint disease activity score using CRP; DMI—deviation from the mean index; DLCO—diffusion capacity for carbon monoxide; FVC—forced vital capacity; HRCT—high-resolution computed tomography; mRSSS—modified Rodnan skin score; RA-ILD—rheumatoid arthritis-associated interstitial lung disease; SSc-ILD—systemic sclerosis-associated interstitial lung disease; 6M—6 months; and 12M—12 months.

Across the entire CTD-ILD cohort, both FVC and DLCO were positively associated with increasing age (r ≈ 0.15–0.36), with the strongest age-related association observed for FVC in the other CTD subgroup (r ≈ 0.52) (Figure 6). Female sex showed weak positive correlations with lung function across most subgroups, particularly for DLCO in RA-ILD (r ≈ 0.42), although these associations were generally modest. In contrast, longer CTD-ILD duration was consistently associated with lower lung function, most prominently for FVC in other CTD-ILDs (r ≈ −0.66), indicating progressive functional impairment with disease chronicity. The correlation heat map identifies radiological disease burden as the most robust and consistently associated determinant of lung function across analyses. Mean ILD extent on HRCT demonstrated moderate-to-strong negative correlations with both FVC and DLCO in nearly all disease groups (r ≈ −0.22 to −0.70), with particularly strong associations for DLCO in SSc-ILD (r ≈ −0.70). Markers of systemic inflammation, reflected by C-reactive protein (CRP) levels, showed weak-to-moderate negative correlations with lung function across several subgroups (approximately r ≈ −0.15 to −0.40). Notably, disease-specific activity indices showed weak and inconsistent associations with lung function parameters across the relevant subgroups. In the RA-ILD subgroup, the baseline DAS28-CRP score demonstrated only a weak negative correlation with FVC (r ≈ −0.24) and a negligible positive correlation with DLCO (r ≈ 0.19). Smoking history was consistently associated with modestly reduced FVC and DLCO across disease groups, although correlations did not reach strong effect sizes. In SSc-ILD, DLCO showed a strong positive correlation with the presence of digital ulcers (r ≈ 0.58, *p* < 0.01), while gastrointestinal (GI) involvement demonstrated a weak positive association with DLCO (r ≈ 0.18). Pulmonary arterial hypertension (PAH) showed a weak negative association with DLCO (r ≈ −0.24), while baseline mRSS score showed a weak negative correlation with FVC (r ≈ −0.34) but a weak positive correlation with DLCO (r ≈ 0.28). In RA-ILD, mean CT disease extent was the dominant determinant of FVC impairment (r ≈ −0.46), whereas DLCO showed weaker and less consistent associations with clinical variables. Other CTD-ILD subgroups demonstrated broadly similar patterns, with lung function primarily driven by radiological extent and disease duration rather than extra-pulmonary manifestations.

### 3.4. Safety and Tolerability of Antifibrotic Therapy

The predominant antifibrotic agent was NTB, constituting the initial therapy for over 95% of the cohort. Most patients (93%) were initiated on NTB monotherapy, while only a small proportion (2.7%) were switched from NTB to PFD (NTD → PFD). The median duration of NTB treatment in our cohort was substantial across all groups (overall: 14.0 months). The other CTD subgroup had the numerically longest treatment duration (17.0 months), although this difference did not reach statistical significance (*p* = 0.123). AEs had a significant impact on treatment delivery, affecting over one-third of the cohort (34.7%). A notably high proportion of patients (39.1%) required a dose reduction, underscoring the need for proactive AE management to enable treatment continuation. Importantly, the frequency of AEs was not uniform across CTD subtypes. A clear gradient is observed, with the SSc subgroup demonstrating the best tolerability profile. Patients with SSc reported the lowest rates of any AE (20.8%), dose reduction (29.1%), and treatment discontinuation (8.3%). In contrast, the other CTD subgroup appeared to have the poorest tolerability, with the highest rates of AEs (45.8%), dose reductions (54.2%), and discontinuations (20.8%). The RA group occupied an intermediate position. While the *p*-values for these comparisons did not reach statistical significance, likely due to the sample size, this consistent trend suggests that the underlying CTD subtype may be an important factor influencing the tolerability of NTB. Among the patients who discontinued treatment (*n* = 12), gastrointestinal AEs were the dominant driver, accounting for the majority of cases. Nausea was the single most common reason (41% of discontinuations), followed by vomiting (16.6%) and diarrhea (16.6%) (Table 4).

## 4. Discussion

In this real-world, two-center Hungarian cohort of patients with CTD-ILD fulfilling contemporary criteria for PPF, antifibrotic treatment—predominantly with NTB—was associated with stabilization or improvement of lung function in most patients, alongside a largely stable or favorable radiologic course over 6–12 months in a routine clinical setting. These findings extend evidence from randomized controlled trials of NTB in progressive fibrosing ILDs and SSc-ILD into a heterogeneous CTD population managed in routine clinical practice [10,25]. Furthermore, we identified distinct patterns of treatment response and tolerability across different CTD subtypes, offering potentially relevant insights for more individualized patient management.

The primary endpoint of our study was radiographic stability, a key therapeutic goal in PPF. More than 70% of patients (71.1% per R1, 78.9% per R2) exhibited either stability or improvement on HRCT after 6–12 months of therapy. This high rate of radiographic non-progression is a significant finding. It aligns with the mechanistic goal of antifibrotics to slow disease progression and provides a tangible, visual correlate to the functional stability measured by FVC [8,11,25]. The observed improvement in GGO in over one-third of patients is particularly noteworthy, as it may reflect resolution of concomitant inflammatory activity, potentially enhanced by background immunosuppressive therapy [26]. In contrast, the stability of traction bronchiectasis—a marker of established fibrosis—supports an effect in halting the progression of irreversible structural damage. Radiographic outcomes remain relatively underreported in the antifibrotic literature [27], where FVC has been the dominant endpoint [28]. Current expert consensus on PPF emphasizes radiographic progression alongside FVC and DLCO as a key marker of disease worsening, and our findings support the use of serial HRCT as a complementary measure of treatment response in CTD-ILD [9].

Our functional data support these radiological findings, demonstrating significant improvements in both FVC and DLCO at 6 and 12 months across the overall cohort. Notably, the rate of clinically meaningful FVC improvement (33.9% at 6 months) exceeds that reported in the landmark INBUILD and SENSCIS trials, where antifibrotic therapy primarily slowed functional decline rather than producing a mean improvement [10,25]. While our findings are broadly consistent with other real-world CTD-ILD cohorts treated with nintedanib [11], this discrepancy likely reflects key differences between clinical trial populations and routine clinical practice. In our cohort, patients were managed within a multidisciplinary framework, where antifibrotic therapy was frequently initiated alongside optimized background immunosuppression. Although most patients remained on stable DMARD or biologic therapy during the observation period, the retrospective design did not allow for comprehensive capture of all treatment modifications, including dose adjustments, treatment intensification, or short-term glucocorticoid use. Therefore, part of the observed functional improvement—particularly in the RA-ILD and other CTD-ILD subgroups—may be attributable to concomitant optimization of ISU therapy or regression to the mean, rather than to antifibrotic treatment alone. While our cohort is comparable in size to several previously published series, it provides a more balanced representation of SSc-ILD, RA-ILD, and other CTD-ILD subtypes and uniquely incorporates a detailed, dual-radiologist HRCT assessment, offering a more granular evaluation of treatment response.

An important observation of our study is the heterogeneity of treatment response across CTD subtypes, which challenges the concept of CTD-ILD as a single therapeutic entity. The RA-ILD subgroup demonstrated the most pronounced functional improvement, with over half (52.9%) showing a >5% increase in FVC at 6 months. This aligns with post hoc analyses of the INBUILD trial, suggesting a particularly favorable response in RA-ILD [27]. The high prevalence of smoking in this group raises the question of whether a combined pulmonary fibrosis and emphysema (CPFE) phenotype or shared pathogenetic mechanisms between smoking-related lung damage and RA-ILD may influence treatment response [29]. The dominant use of methotrexate and rituximab reflects standard RA management and may contribute to the observed outcomes through combined antifibrotic and immunomodulatory effects [30]. In contrast, the SSc-ILD subgroup exhibited a pattern characterized predominantly by stability. The significant within-group improvement in FVC and the shift in patients into higher FVC % predicted categories are clinically meaningful outcomes. This finding is directly consistent with the SENSCIS trial, which demonstrated that NTB reduces the rate of FVC decline in SSc-ILD [25], supporting the external validity of trial data in routine practice. The other CTD-ILD group, while heterogeneous, also showed significant functional improvements, particularly in DLCO. This group, which included patients with IIM and AAV, often required more aggressive immunosuppression (evidenced by the high use of cyclophosphamide and combination therapies), suggesting that NTB may provide a crucial antifibrotic backbone in complex, treatment-refractory cases [9].

From a therapeutic standpoint, our cohort reflects evolving practice patterns toward combination strategies [8]. While many patients had received conventional or biologic immunosuppression sequentially before NTB, a substantial proportion were on concomitant therapy at the time of evaluation, including combinations of MMF with rituximab or tocilizumab. This mirrors other CTD-ILD series where most patients (80–98%) received NTB as add-on therapy [31]. Such an approach is aligned with the recently published ERS/EULAR guidelines, which suggest the use of NTB in patients with CTD-ILD and PPF, preferably in combination with appropriate immunosuppressive therapy [9]. Our findings support that this combined therapeutic approach is feasible across diverse CTD phenotypes and may be associated with stabilization of both lung function and radiologic involvement in real-world care.

Correlation analyses provided additional context. Across CTD-ILD, SSc-ILD, RA-ILD, and other CTD-ILD subgroups, the extent of ILD on HRCT was the most robust correlate of both FVC and DLCO, reinforcing radiologic disease burden as a key determinant of functional impairment. This echoes post hoc analyses of SENSCIS, where greater fibrotic extent was associated with more pronounced FVC decline and where NTB reduced progression irrespective of baseline extent [32]. Our multivariate analysis confirms that while the radiological extent of ILD is a universally strong predictor of functional decline, the contribution of demographic, serological, and extra-pulmonary clinical manifestations varies significantly across different CTDs, underscoring the need for a tailored approach to management and prognostication. Given the small sample sizes in subgroup analyses—particularly the heterogeneous “other CTD” group (*n* = 24, encompassing several rare disease entities)—all correlation findings should be considered exploratory and hypothesis-generating rather than definitive or mechanistic. With this caveat, we observed several associations that merit further investigation. For example, in the SSc-ILD subgroup, there was a positive correlation between DLCO and digital ulcers (β = +0.58). While counterintuitive, this finding raises the hypothesis that patients with prominent peripheral vasculopathy may receive more intensive vasoactive therapy (e.g., phosphodiesterase-5 inhibitors or endothelin receptor antagonists), which could coincidentally benefit pulmonary gas exchange [33]. In contrast, the expected negative correlation between DLCO and pulmonary arterial hypertension (β = −0.24) was also observed. In the “other CTD” group, the negative correlation between ILD duration and FVC (β = −0.66) suggests a possible cumulative, time-dependent lung injury, supporting the importance of early detection. Similarly, the negative correlation between CRP and DLCO (β = −0.40) in this group raises the hypothesis that systemic inflammation may contribute to gas exchange impairment, but this requires confirmation in larger, dedicated cohorts.

Our safety analysis reveals a critical nuance: the tolerability of NTB is not uniform and may vary across CTD subtypes. The SSc-ILD group demonstrated a more favorable tolerability profile, whereas higher rates of adverse events and dose modifications were observed in the other CTD-ILD group. Although limited by sample size, these findings are clinically plausible and underscore the importance of individualized treatment strategies. Patients with SSc are often younger, have longer-standing diagnoses, and may be more accustomed to managing chronic symptoms, including gastrointestinal (GI) issues, which are also common in SSc itself [34]. Conversely, patients in the other CTD group may have more systemic inflammatory burden or comorbidities that lower their threshold for tolerating drug-related AEs. The high rate of dose reductions (39.1% overall) underscores the importance of proactive management, patient education, and dose titration to ensure long-term treatment persistence, as recommended in clinical guidelines [9,35,36].

Our study has several strengths. It represents, to our knowledge, one of the larger real-world CTD-ILD cohorts treated with NTB in Central and Eastern Europe, includes a broad spectrum of CTDs, and integrates detailed clinical, functional, and radiologic data assessed by two independent radiologists. The strict application of contemporary PPF and national reimbursement criteria ensures that our findings are relevant to current clinical practice and guideline-based indications [9].

Certain limitations of our study must be acknowledged. Its retrospective and observational design introduces the potential for selection and information bias. The sample size, while substantial for a real-world cohort, limits the statistical power for subgroup analyses, particularly for the rare CTDs within the other CTD group. Other composite disease activity scores, such as the Simplified Disease Activity Index (SDAI) or Clinical Disease Activity Index (CDAI), were not available, and detailed longitudinal glucocorticoid dosing data were also lacking (only 12.5% of patients received glucocorticoids, and therapy was inconsistent over time), preventing a robust assessment of glucocorticoid-sparing effects. The lack of a matched control group of CTD-ILD patients not receiving antifibrotic therapy prevents a direct comparative estimate of treatment effect size. Furthermore, without a control arm, we cannot fully exclude that some of the observed functional improvement may be attributable to regression to the mean or to concurrent optimization of background immunosuppression, although the latter remained largely stable during the study period. Finally, the relatively short follow-up period of 12–24 months captures medium-term outcomes but cannot assess the long-term sustainability of the observed benefits.

## 5. Conclusions

In conclusion, this real-world study from Central and Eastern Europe provides supportive evidence for the use of NTB in patients with progressive fibrosing CTD-ILD. Our findings suggest that treatment is associated with a high rate of radiological and functional stability, and even improvement, in a routine care setting. Our findings reinforce the central role of antifibrotic therapy in the management of PPF, as endorsed by the recent ERS/EULAR consensus [9], and highlight the importance of a multidisciplinary approach [37]. The observed heterogeneity in treatment response and tolerability across CTD subtypes calls for further large-scale, prospective studies to better identify predictors of response and to refine personalized treatment algorithms that optimally integrate immunosuppressive and antifibrotic strategies.

## Figures and Tables

**Table 1:** Baseline characteristics of the study population.

Major Subgroups of Patients with ^1^ CTD					
Characteristics	Total CTD Patients *n* = 72 (100%)	SSc *n* = 25 (34.7%)	RA *n* = 23 (31.9%)	Other CTD Groups *n* = 24 (33.3%)	*p*-Value
Demographics					
Age, years	64.5 (57.0–68.0)	62.0 (56.0–67.0)	65.0 (61.0–68.0)	65.0 (54.0–71.5)	0.519
Sex, female	51 (70.8%)	20 (80.0%)	13 (56.5%)	18 (75.0%)	0.174
Exposition and life-style					
Environmental and occupational exposure	23 (31.9%)	7 (28.0%)	9 (39.1%)	7 (29.2%)	0.667
Smoking status					**<0.001**
Never	41 (56.9%)	18 (72.0%) ^2^	6 (26.1%) ^1,3^	17 (70.8%) ^2^	
Past	23 (31.9%)	6 (24.0%)	10 (43.5%)	7 (29.2%)	
Current	8 (11.1%)	1 (4.0%)	7 (30.4%)	0 (0.0%)	
Disease characteristics					
Duration of ^1^ CTD	8.5 (4.0–18.0)	13.0 (7.0–22.0) ^3^	7.0 (3.0–16.0)	5.5 (2.5–15.5) ^1^	**0.044**
Duration of ^2^ CTD-ILD	3.5 (1.0–7.0)	6.0 (3.0–12.0) ^2^	1.0 (0.0–4.0) ^1^	2.5 (1.0–9.0)	**0.019**
Autoantibody profile					
RF positivity	23 (31.9%)	1 (4.0%) ^2^	18 (78.3%) ^1,3^	4 (16.7%) ^2^	**<0.001**
Anti-CCP positivity	17 (23.6%)	0 (0%) ^2^	16 (69.6%)^1,3^	1 (4.2%) ^2^	**<0.001**
Anti-Scl70 positivity	17 (23.6%)	15 (60%) ^2,3^	0 (0%) ^1^	2 (8.3%) ^1^	**<0.001**
Anti-Ro52 positivity	16 (22.2%)	4 (16.0%)	3 (13.0%)	9 (37.5%)	0.085
Anti-Jo1 positivity	4 (5.6%)	0 (0%)^3^	0 (0%)^3^	4 (16.7%) ^1,2^	**0.019**
Anti-Pl7 positivity	1 (1.4%)	0 (0%)	0 (0%)	1 (4.2%)	**0.653**
Disease activity at baseline					
DAS28-CRP score	N.A.	N.A.	3.2 (2.4–4.9)	N.A.	
mRSSS	N.A.	8.0 (4.0–12.0)	N.A.	N.A.	
Previous immunosuppressive agents					
Hydroxychloroquine	12 (16.7%)	3 (12.0%)	4 (17.4%)	5 (20.8%)	0.733
Glucocorticoids	16 (22.2%)	3 (12.0%)	6 (26.1%)	7 (29.2%)	0.304
Azathioprine	9 (12.5%)	4 (16.0%)	0 (0.0%)	5 (20.8%)	0.060
Methotrexate	23 (31.9%)	4 (16.0%) ^2^	15 (65.2%) ^1,3^	4 (16.7%) ^2^	**<0.001**
Mycophenolate mofetil	22 (30.6%)	12 (48.0%) ^2^	1 (4.3%) ^1,3^	9 (37.5%) ^2^	**0.003**
Cyclophosphamide	28 (38.9%)	13 (52.0%) ^2^	1 (4.3%) ^1,3^	14 (58.3%) ^2^	**<0.001**
Sulphasalazine	7 (9.7%)	1 (4.0%) ^2^	6 (26.1%) ^1,3^	0 (0.0%) ^2^	**0.004**
Leflunomide	2 (2.8%)	0 (0.0%)	2 (8.7%)	0 (0.0%)	0.099
Cyclosporin A	4 (5.6%)	0 (0.0%) ^3^	0 (0.0%)	4 (16.7%) ^1^	**0.019**
Rituximab	17 (23.6%)	2 (8.0%) ^2^	9 (39.1%) ^1^	6 (25.0%)	**0.039**
Tocilizumab	13 (18.1%)	4 (16.0%)	3 (13.0%)	6 (25.0%)	0.553
TNF inhibitor	3 (4.2%)	0 (0.0%)	3 (13.0%)	0 (0.0%)	**0.030**
JAK inhibitor	2 (2.8%)	0 (0.0%)	2 (8.7%)	0 (0.0%)	0.099
None	5 (6.9%)	4 (16.0%)	0 (0.0%)	1 (4.2%)	0.119
Concomitant immunosuppressive agents					
Glucocorticoids	16 (22.2%)	3 (12%)	6 (26.1%)	7 (29.2%)	0.304
Mycophenolate mofetil	13 (18.1%)	9 (36.0%) ^2^	0 (0.0%) ^1^	4 (16.7%)	**0.003**
Rituximab	11 (15.3%)	0 (0.0%) ^2^	7 (30.4%) ^1^	4 (16.7%)	**0.006**
Rituximab + Mycophenolate mofetil	5 (6.9%)	2 (8.0%)	1 (4.3%)	2 (8.3%)	>0.999
Rituximab + Methotrexate	3 (4.2%)	0 (0.0%)	3 (13.0%)	0 (0.0%)	**0.030**
Tocilizumab	5 (6.9%)	0 (0.0%)	3 (13.0%)	2 (8.3%)	0.154
Tocilizumab + Mycophenolate mofetil	5 (6.9%)	2 (8.0%)	0 (0.0%)	3 (12.5%)	0.314
Tocilizumab + Methotrexate	1 (1.4%)	1 (4.0%)	0 (0.0%)	0 (0.0%)	>0.999
None	29 (40.3%)	11 (44.0%)	9 (39.1%)	9 (37.5%)	0.890

Note: Data are presented as *n* (%) or median (IQR). Bold *p*-values are statistically significant (*p* < 0.05). ^1,2,3^ Post hoc analysis indicates the value is significantly different from the SSc-ILD (1), RA-ILD (2), or other CTD-ILD (3) group, respectively. Abbreviations: CTD—connective tissue disease; SSc—systemic sclerosis; RA—rheumatoid arthritis; RF—rheumatoid factor; anti-CCP—anti-cyclic citrullinated peptide; anti-Scl70—anti-topoisomerase I; anti-Ro52—anti-tripartite motif-containing protein 21; anti-Jo1—anti–histidyl-tRNA synthetase; anti-Pl7—anti-threonyl-tRNA synthetase; DAS28-CRP—28 joint disease activity score using CRP; mRSSS—modified Rodnan skin score.

**Table 2:** Impact of antifibrotic therapy on pulmonary function.

	Total CTD Patients (*n* = 72)			SSc (*n* = 25)			RA (*n* = 23)			Other CTD Groups (*n* = 24)		
Characteristics	0	6M	12M	0	6M	12M	0	6M	12M	0	6M	12M
FVC	*n* = 67	*n* = 57	*n* = 37	*n* = 22	*n* = 17	*n* = 10	*n* = 21	*n* = 18	*n* = 12	*n* = 24	*n* = 22	*n* = 15
FVC (%)	85.6 ± 23.3	88.9 ± 22.7	88.9 ± 23.5	89.2 ± 18.3	89.6 ± 15.9	96.3 ± 24.5	79.6 ± 25.6	90.2 ± 26.0	83.3 ± 29.2	87.6 ± 25.1	87.3 ± 25.1	88.4 ± 17.3
FVC (%) category												
>80%	37 (55.2%)	39 (68.4%)	24 (64.9%)	13 (59.1%)	13 (76.5%)	7 (70.0%)	9 (42.9%)	13 (72.2%)	8 (66.7%)	15 (62.5%)	13 (59.1%)	9 (60.0%)
>70%	13 (19.4%)	8 (14.0%)	4 (10.8%)	6 (27.3%)	2 (11.8%)	1 (10.0%)	4 (19.0%)	1 (5.6%)	0 (0.0%)	3 (12.5%)	5 (22.7%)	3 (20.0%)
50–70%	14 (20.9%)	6 (10.5%)	7 (18.9%)	3 (13.6%)	2 (11.8%)	2 (20.0%)	7 (33.3%)	2 (11.1%)	2 (16.7%)	4 (16.7%)	2 (9.1%)	3 (20.0%)
<50%	3 (4.5%)	4 (7.0%)	2 (5.4%)	0 (0.0%)	0 (0.0%)	0 (0.0%)	1 (4.8%)	2 (11.1%)	2 (16.7%)	2 (8.3%)	2 (9.1%)	0 (0.0%)
Change in FVC (%) *		***p *** **< 0.001**	***p *** **< 0.001**		***p *** **< 0.001**	***p *** **= 0.100**		***p *** **= 0.047**	***p *** **= 0.150**		***p *** **= 0.062**	***p *** **= 0.022**
>5% improvement (1)		19 (33.9%) ^3^	8 (23.5%) ^2^		3 (17.6%) ^2^	2 (22.2%)		9 (52.9%) ^3^	3 (30.0%)		7 (31.8%)	3 (20.0%)
stable (2)		33 (58.9%) ^3^	22 (64.7%) ^1,3^		14 (82.4%) ^1,3^	6 (66.7%)		7 (41.2%) ^3^	6 (60.0%)		12 (54.5%)	10 (66.7%) ^3^
>5% decline (3)		4 (7.1%) ^1,2^	4 (11.8%) ^2^		0 (0.0%) ^2^	1 (11.1%)		1 (5.9%) ^1,2^	1 (10.0%)		3 (13.6%)	2 (13.3%) ^2^
DLCO	*n* = 65	*n* = 56	*n* = 35	*n* = 22	*n* = 16	*n* = 9	*n* = 19	*n* = 17	*n* = 11	*n* = 24	*n* = 23	*n* = 15
DLCO (%)	59.2 ± 19.8	62.8 ± 18.6	61.7 ± 22.9	64.8 ± 22.6	64.6 ± 17.8	63.2 ± 20.0	56.8 ± 17.6	65.6 ± 16.6	65.0 ± 24.0	55.9 ± 18.3	59.4 ± 20.7	58.4 ± 24.7
DLCO (%) category												
>75%	12 (18.5%)	13 (23.2%)	7 (20.0%)	5 (22.7%)	4 (25.0%)	1 (11.1%)	4 (21.1%)	4 (23.5%)	3 (27.3%)	3 (12.5%)	5 (21.7%)	3 (20.0%)
>60%	14 (21.5%)	19 (33.9%)	10 (28.6%)	6 (27.3%)	6 (37.5%)	4 (44.4%)	4 (21.1%)	7 (41.2%)	3 (27.3%)	4 (16.7%)	6 (26.1%)	3 (20.0%)
40–60%	30 (46.2%)	14 (25.0%)	13 (37.1%)	10 (45.5%)	4 (25.0%)	3 (33.3%)	6 (31.6%)	4 (23.5%)	3 (27.3%)	14 (58.3%)	6 (26.1%)	7 (46.7%)
<40%	9 (13.8%)	10 (17.9%)	5 (14.3%)	1 (4.5%)	2 (12.5%)	1 (11.1%)	5 (26.3%)	2 (11.8%)	2 (18.2%)	3 (12.5%)	6 (26.1%)	2 (13.3%)
Change in DLCO (%) *		***p *** **< 0.001**	***p *** **< 0.001**		***p *** **= 0.002**	***p *** **= 0.044**		***p *** **= 0.015**	***p *** **= 0.002**		***p *** **= 0.054**	***p *** **< 0.001**
>10% improvement (1)		15 (27.3%) ^2^	5 (15.6%) ^2^		2 (12.5%) ^2^	1 (12.5%) ^2^		7 (43.8%) ^3^	1 (11.1%) ^2^		6 (26.1%)	3 (20.0%) ^2^
stable (2)		34 (61.8%) ^1,3^	26 (81.3%) ^1,3^		12 (75.0%) ^1,3^	6 (75.0%) ^1,3^		9 (56.3%) ^3^	8 (88.9%) ^1,3^		13 (56.5%)	12 (80.0%) ^1,3^
>10% decline (3)		6 (10.9%) ^2^	1 (3.1%) ^2^		2 (12.5%) ^2^	1 (12.5%) ^2^		0 (0.0%) ^1,2^	0 (0.0%) ^2^		4 (17.4%)	0 (0.0%) ^2^

Notes: Data are presented as *n* (%) or mean ± SD. * Calculation of FVC (%) and DLCO (%) change: For the 6-month time point, the reference value is the pre-treatment value. For the 12-month time point, the reference value is the 6-month value. Bold *p*-values indicate a statistically significant result (*p* < 0.05). ^1,2,3^ The given change category is significantly different from the >5% or >10% improvement category (1), the stable category (2), and the >5% or >10% decline category (3). Abbreviations: CTD—connective tissue disease; SSc—systemic sclerosis; RA—rheumatoid arthritis; 6M—6 months; 12M—12 months; FVC—forced vital capacity; and DLCO—diffusion capacity for carbon monoxide.

**Table 3:** Treatment response based on the HRCT assessments of the two radiologists.

Response to Therapy	Radiologist 1	Radiologist 2	Difference	Cohen’s κ
		GGO (*n* = 29)		0.32
No change	9 (31.0%)	9 (31.0%)	0%	
Minimal improvement	6 (20.7%)	5 (17.2%)	+3.5% R1	
Moderate/marked improvement	4 (13.8%)	6 (20.7%)	+6.9% R2	
Minimal deterioration	9 (31.0%)	7 (24.1%)	+6.9% R1	
Moderate/marked deterioration	1 (3.4%)	2 (6.9%)	+3.5% R2	
	Bronchiectasis (*n* = 37)			0.11
No change	25 (67.6%)	28 (75.7%)	+8.1% R2	
Minimal improvement	5 (13.5%)	7 (18.9%)	+5.4% R2	
Moderate/marked improvement	1 (2.7%)	0 (0.0%)	+2.7% R1	
Minimal deterioration	6 (16.2%)	2 (5.4%)	+10.8% R1	
	Overall therapeutic response (*n* = 38)			0.36
No change	13 (34.2%)	18 (47.4%)	+13.2% R2	
Minimal improvement	10 (26.3%)	7 (18.4%)	+7.9% R1	
Moderate/marked improvement	4 (10.5%)	5 (13.2%)	+2.7% R2	
Minimal deterioration	7 (18.4%)	6 (15.8%)	+2.6% R1	

Notes: Data are presented as *n* (%). κ values: poor (<0), slight (0.00–0.20), fair (0.21–0.40), moderate (0.41–0.60), substantial (0.61–0.80), and almost perfect (0.81–1.00). ICC values: poor (<0.50), moderate (0.50–0.74), good (0.75–0.89), and excellent (≥0.90). Abbreviations: HRCT—high-resolution computed tomography; GGO—ground-glass opacity; R1—radiologist 1; and R2—radiologist 2.

**Table 4:** Baseline characteristics, treatment patterns, and outcomes in CTD-ILD patients treated with antifibrotics.

Major Subgroups of Patients with CTD					
Characteristics	Total CTD Patients *n* = 72 (100%)	SSc *n* = 25 (34.7%)	RA *n* = 23 (31.9%)	Other CTD Groups *n* = 24 (33.3%)	*p*-Value
Type of antifibrotic regimen (NTB/PFD)					0.225
NTB only	67 (93%)	22 (88%)	21 (95.7%)	24 (100%)	
NTB → PFD	2 (2.7%)	2 (8%)	0 (0%)	0 (0%)	
PFD only	3 (4.2%)	1 (4%)	2 (8.7%)	0 (0%)	
Treatment duration (months)					
NTB	14.0 (7.0–27.0)	14.0 (8.0–32.0)	12.0 (5.0–19.0)	17.0 (9.0–35.0)	0.123
PFD	12.5 (4.0–19.0)	11.3 (3.0–19.0)	9.5 (4.0–15.0)	-	n.c.
Impact of adverse events on NTB treatment					
Adverse events (total)	24 (34.7%)	5 (20.8%)	8 (38.1%)	11 (45.8%)	0.178
Dose reduction	27 (39.1%)	7 (29.1%)	7 (33.3%)	13 (54.2%)	0.167
Discontinuation	12 (17.3%)	2 (8.3%)	5 (23.8%)	5 (20.8%)	0.378
Reason for discontinuation					n.c.
Headache/Hypertensive crisis	1 (8.3%)	0 (0%)	0 (0%)	1 (20%)	
Nausea	5 (41%)	1 (50%)	2 (40%)	2 (40%)	
Vomiting	2 (16.6%)	0 (0%)	1 (20%)	1 (20%)	
Abdominal pain	1 (8.3%)	0 (0%)	0 (0%)	1 (20%)	
Diarrhea	2 (16.6%)	1 (50%)	1 (20%)	0 (0%)	
Elevated liver enzymes	1 (8.3%)	0 (0%)	1 (20%)	0 (0%)	

Note: Data are presented as *n* (%). Abbreviations: CTD-ILD—connective tissue disease-associated interstitial lung disease; CTD—connective tissue disease; SSc—systemic sclerosis; RA—rheumatoid arthritis; NTB—nintedanib; PFD—pirfenidone; and n.c.—not calculated.

## Data Availability

The data presented in this study are available on request from the corresponding author.

## Supplementary Materials

The following supporting information can be downloaded at: https://www.mdpi.com/article/10.3390/jcm15093539/s1; Table S1: Baseline characteristics of the CTD-ILD study cohort by disease subtype.

## Abbreviations

The following abbreviations are used in this manuscript:

AAV	Anti-neutrophil cytoplasmic antibody vasculitis
ACR	American College of Rheumatology
AE	Adverse event
ALAT	Latin American Thoracic Association
ANCA	Anti-neutrophil cytoplasmic antibody
Anti-CCP	Anti-cyclic citrullinated peptide
Anti-Jo1	Anti-histidyl-tRNA synthetase
Anti-Pl7	Anti-threonyl-tRNA synthetase
Anti-Scl70	Anti-topoisomerase I
ATS	American Thoracic Society
CHP	Chronic hypersensitivity pneumonitis
CPFE	Combined pulmonary fibrosis and emphysema
CRP	C-reactive protein
CT	Computed tomography
CTD-ILD	Connective tissue disease-associated interstitial lung disease
CYC	Cyclophosphamide
DAS28-CRP	28 joint disease activity score using CRP
DLCO	Diffusing capacity for carbon monoxide
ERS	European Respiratory Society
EULAR	European Alliance of Associations for Rheumatology
fNSIP	Fibrotic non-specific interstitial pneumonia
FVC	Forced vital capacity
GGO	Ground-glass opacity
GI	Gastrointestinal
HC	Honeycombing
HRCT	High-resolution computed tomography
ICC	Intraclass correlation coefficient
ILD	Interstitial lung disease
IIM	Idiopathic inflammatory myopathies
IPAF	Interstitial pneumonia with autoimmune features
IQR	Interquartile range
JRS	Japanese Respiratory Society
MCTD	Mixed connective tissue disease
MDD	Multidisciplinary discussion
MMF	Mycophenolate mofetil
mRSSS	Modified Rodnan skin score
MTX	Methotrexate
NSIP	Non-specific interstitial pneumonia
NTB	Nintedanib
OP	Organizing pneumonia
PAH	Pulmonary arterial hypertension
PFD	Pirfenidon
PFT	Pulmonary function test
PPF	Progressive pulmonary fibrosis
RA	Rheumatoid arthritis
RF	Rheumatoid factor
Ro-52	Tripartite motif-containing protein 21
RTX	Rituximab
SD	Standard deviation
SjD	Sjögren’s disease
SLE	Systemic lupus erythematosus
SSc	Systemic sclerosis
TCZ	Tocilizumab
TLV	Total lung volume
UCTD	Undifferentiated connective tissue disease
UIP	Usual interstitial pneumonia
