# Supplementary material for: Real-World Data on Halting Radiographic Progression with Antifibrotics in Connective Tissue Disease-Associated Interstitial Lung Disease: A Two-Center Study from Hungary
Source: J Clin Med. 2026 May 6;15(9):3539. doi: 10.3390/jcm15093539 (PMC13164095; doi:10.3390/jcm15093539)
Supplement: Supplementary file 1 [file jcm-15-03539-s001.zip › jcm-4274191-supplementary.pdf]

Supplementary Material

Table S1. Baseline characteristics of the CTD-ILD study cohort by disease subtype.

	Additional Subgroups of Patients with CTD					
**Characteristics**	**IPAF** *n* = 6 (25%)	**IIM** *n* = 5 (20.8%)	**AAV** *n* = 3 (12.5%)	**SjD** *n* = 3 (12.5%)	**MCTD** *n* = 2 (8.3%)	**Overlap** *n* = 5 (20.8%)
Demographics						
Age, years	67.5 (63–71)	51 (49–52)	72 (64–74)	74 (66–84)	65.5 (65–66)	55 (53–60)
Sex, female	4 (66.7%)	4 (80%)	2 (66.7%)	2 (66.7%)	2 (100%)	4 (80%)
Exposition and lifestyle						
Environmental and occupational exposure	3 (50%)	0 (0%)	0 (0%)	1 (33.3%)	1 (50%)	2 (40%)
Smoking status						
Never	4 (66.7%)	5 (100%)	2 (66.7%)	2 (66.7%)	1 (50%)	3 (60%)
Past	2 (33.3%)	0 (0%)	1 (33.3%)	1 (33.3%)	1 (50%)	2 (40%)
Current	0 (0%)	0 (0%)	0 (0%)	0 (0%)	0 (0%)	0 (0%)
Disease characteristics						
Duration of CTD	2 (2–3)	16 (15–20)	6 (2–11)	8 (1–8)	17.5 (4–31)	5 (4–17)
Duration of CTD-ILD	1.5 (1–2)	17 (6–20)	0 (0–5)	0.5 (0–1)	8 (4–12)	4 (3–14)
Autoantibody profile						
RF positivity	1 (16.7%)	0 (0%)	0 (0%)	1 (33.3%)	0 (0%)	2 (40%)
Anti-CCP positivity	0 (0%)	0 (0%)	0 (0%)	0 (0%)	0 (0%)	1 (20%)
Anti-Scl70 positivity	1 (16.7%)	0 (0%)	0 (0%)	0 (0%)	0 (0%)	1 (20%)
Anti-Ro52 positivity	4 (66.7%)	1 (20%)	0 (0%)	3 (100%)	0 (0%)	1 (20%)
Anti-Jo1 positivity	0 (0%)	2 (40%)	0 (0%)	0 (0%)	0 (0%)	2 (40%)
Anti-Pl7 positivity	0 (0%)	1 (20%)	0 (0%)	0 (0%)	0 (0%)	0 (0%)
Previous immunosuppressive agents						
Hydroxychloroquine	1 (16.7%)	1 (20%)	0 (0%)	2 (66.7%)	0 (0%)	1 (20%)
Glucocorticoids	2 (33.3%)	1 (20%)	1 (33.3%)	1 (33.3%)	0 (0%)	2 (40%)
Azathioprine	0 (0%)	1 (20%)	1 (33.3%)	1 (33.3%)	1 (50%)	1 (20%)
Methotrexate	1 (16.7%)	0 (0%)	0 (0%)	0 (0%)	1 (50%)	2 (40%)
Mycophenolate mofetil	5 (83.3%)	1 (20%)	0 (0%)	0 (0%)	1 (50%)	2 (40%)
Cyclophosphamide	3 (50%)	4 (80%)	2 (66.7%)	0 (0%)	1 (50%)	4 (80%)
Cyclosporin A	0 (0%)	2 (40%)	0 (0%)	0 (0%)	0 (0%)	2 (40%)
Rituximab	0 (0%)	1 (20%)	1 (33.3%)	0 (0%)	0 (0%)	4 (80%)
Tocilizumab	2 (33.3%)	1 (20%)	0 (0%)	0 (0%)	1 (50%)	2 (40%)
None	0 (0%)	0 (0%)	1 (33.3%)	0 (0%)	0 (0%)	0 (0%)
Concomitant immunosuppressive agents						
Glucocorticoids	2(33.3%)	2 (40%)	2 (66.7%)	1 (33.3%)	0 (0%)	0 (0%)
Mycophenolate mofetil	4 (66.7%)	0 (0%)	0 (0%)	0 (0%)	0 (0%)	0 (0%)
Rituximab	0 (0%)	0 (0%)	2 (66.7%)	0 (0%)	0 (0%)	2 (40%)
Rituximab + Mycophenolate mofetil	0 (0%)	1 (20%)	0 (0%)	0 (0%)	0 (0%)	1 (20%)
Tocilizumab	1 (16.7%)	0 (0%)	0 (0%)	0 (0%)	1 (50%)	0 (0%)
Tocilizumab + Mycophenolate mofetil	1 (16.7%)	1 (20%)	0 (0%)	0 (0%)	0 (0%)	1 (20%)
None	0 (0%)	3 (60%)	1 (33.3%)	3 (100%)	1 (50%)	1 (20%)

Abbreviations: AAV—antineutrophilic cytoplasmic antibody-associated vasculitis; CTD—connective tissue disease; CTD-ILD—connective tissue disease-associated interstitial lung disease; IPAF—interstitial pneumonia with autoimmune features; IIM—idiopathic inflammatory myopathies; MCTD—mixed connective tissue disease; and SjD—Sjögren's disease.
